# Vitamin D and the epigenome

**DOI:** 10.3389/fphys.2014.00164

**Published:** 2014-04-29

**Authors:** Irfete S. Fetahu, Julia Höbaus, Enikő Kállay

**Affiliations:** Department of Pathophysiology and Allergy Research, Center of Pathophysiology, Infectiology and Immunology, Comprehensive Cancer Center, Medical University of ViennaVienna, Austria

**Keywords:** VDR, VDRE, 1,25-dihydroxyvitamin D3, CYP27B1, CYP24A1, DNA methylation, histone modifications, CpG island

## Abstract

Epigenetic mechanisms play a crucial role in regulating gene expression. The main mechanisms involve methylation of DNA and covalent modifications of histones by methylation, acetylation, phosphorylation, or ubiquitination. The complex interplay of different epigenetic mechanisms is mediated by enzymes acting in the nucleus. Modifications in DNA methylation are performed mainly by DNA methyltransferases (DNMTs) and ten-eleven translocation (TET) proteins, while a plethora of enzymes, such as histone acetyltransferases (HATs), histone deacetylases (HDACs), histone methyltransferases (HMTs), and histone demethylases (HDMs) regulate covalent histone modifications. In many diseases, such as cancer, the epigenetic regulatory system is often disturbed. Vitamin D interacts with the epigenome on multiple levels. Firstly, critical genes in the vitamin D signaling system, such as those coding for vitamin D receptor (*VDR*) and the enzymes 25-hydroxylase (*CYP2R1*), 1α-hydroxylase (*CYP27B1*), and 24-hydroxylase (*CYP24A1*) have large CpG islands in their promoter regions and therefore can be silenced by DNA methylation. Secondly, VDR protein physically interacts with coactivator and corepressor proteins, which in turn are in contact with chromatin modifiers, such as HATs, HDACs, HMTs, and with chromatin remodelers. Thirdly, a number of genes encoding for chromatin modifiers and remodelers, such as HDMs of the Jumonji C (JmjC)-domain containing proteins and lysine-specific demethylase (LSD) families are primary targets of VDR and its ligands. Finally, there is evidence that certain VDR ligands have DNA demethylating effects. In this review we will discuss regulation of the vitamin D system by epigenetic modifications and how vitamin D contributes to the maintenance of the epigenome, and evaluate its impact in health and disease.

## Introduction

The role of vitamin D in regulating gene expression has become increasingly evident since the discovery of the transcription factor vitamin D receptor (VDR), a member of the steroid nuclear receptor superfamily. The effect of liganded VDR depends on the epigenetic landscape of the target gene. Genome wide analysis in the human leukemia cell line THP-1 showed that VDR binds mainly at loci of open chromatin. Upon treatment with the VDR ligand 1,25-dihydroxyvitamin D_3_ (1,25-D_3_), chromatin accessibility further increases in more than 30% of these loci (Seuter et al., 2013). The mechanism of action of the liganded VDR is dependent on binding and action of histone acetyltransferases (HATs) and histone methyltransferases (HMTs). It has been shown that co-treatment of cells with 1,25-D_3_, and histone deacetylase or DNA methyltransferase inhibitors often have synergistic effects (Pan et al., 2010).

Many common diseases have both genetic and epigenetic components, which communicate in an intricate and multilayered manner. Currently, it is not clear to what extent epigenetic alterations contribute to onset and progress of common diseases, such as cancer. Epigenetics refers to processes that alter gene activity without changing the DNA sequence. They play an important role in regulating key processes during development, including embryonic developmental events, gene imprinting, and inactivation of chromosome X in females (Bird, 2002; Meissner et al., 2008; Tsai and Baylin, 2011). Maintenance of normal functioning of these biological processes is dependent on the intricate interaction between several epigenetic mechanisms, such as DNA methylation, histone modifications, and non-coding RNAs (Jones and Baylin, 2007). Therefore, at a given promoter the marks arising from DNA methylation and histone modifications determine whether the chromatin is in an open (active) or a closed (repressed) state. Deregulation of the epigenetic mechanisms can lead to aberrant DNA methylation patterns and chromatin architecture, which is a common feature in cancer (Baylin and Jones, 2011; Tsai and Baylin, 2011; Helin and Dhanak, 2013).

## Epigenetic changes mediated by the vitamin D receptor and its ligands

The effect of nutrition on the methylation equilibrium of the genome is already accepted as one of the mechanisms preventing either promoter hyper- or global hypomethylation. Several nutrients are renowned for their impact on DNA methylation, such as folic acid, vitamin B, green tea, and alcohol (Arasaradnam et al., 2008). The effect of vitamin D is currently under debate.

Primary epigenetic effects of vitamin D are linked to histone modifications, mainly acetylation. The VDR/RXR dimer interacts with HATs to induce transcriptional activation (Karlic and Varga, 2011). Several studies have suggested that vitamin D may affect also DNA methylation. A recent study associated severe vitamin D deficiency with methylation changes in leukocyte DNA, although the observed differences were relatively small (Zhu et al., 2013). This study suggested that subjects with vitamin D deficiency were more likely to show reduced synthesis and increased catabolism of active vitamin D. Whether this was the cause of the vitamin D deficiency or the consequence thereof is not clear and needs further studies.

### Effect of vitamin D on DNA methylation

DNA methylation is the most extensively studied epigenetic mark (Esteller, 2008). In humans, DNA methylation occurs on cytosines followed by guanine (CpG) (Bird, 1980; Gruenbaum et al., 1981). Regions of DNA enriched in CpG clusters form CpG islands (CGI) (Wang and Leung, 2004). DNA methylation is necessary for regulating and orchestrating key biological processes, including cell cycle, differentiation, as well as genomic imprinting (Feinberg et al., 2002; Reik and Lewis, 2005; Jones and Baylin, 2007). DNA hypermethylation is mainly found in intergenic regions and repetitive genomic sequences to maintain these in a transcriptionally inactive chromatin state (Herman and Baylin, 2003).

DNA methyltransferases (DNMTs) mediate DNA methylation (Robertson, 2005). DNMT1 encodes for a maintenance methyltransferase, whereas DNTM3A/3B encode for de novo methyltransferases, which are pivotal to maintain and establish genomic methylation (Okano et al., 1998, 1999; Jin and Robertson, 2013). However, *in vivo* studies suggest that all three DNMTs might exert both de novo and maintenance functions (Rhee et al., 2000, 2002; Kim et al., 2002; Esteller, 2007a). Recently, a new group of enzymes that induce active demethylation of the DNA was discovered, the ten-eleven translocation (TET) enzyme family, which plays an important role both in development and tumorigenesis (Kriaucionis and Heintz, 2009; Ficz et al., 2011; Williams et al., 2011; Yamaguchi et al., 2012; Hackett et al., 2013).

Alterations in the cancer epigenome are generally associated with loss of global DNA methylation and gain of methylation in specific gene promoters (Ting et al., 2006). Loss of global methylation may lead to chromosomal instability (Eden et al., 2003), loss of imprinting (Cui et al., 2003; Bjornsson et al., 2007), and activation of transposable elements, thereby leading to disturbances in the genome (Bestor, 2005; Esteller, 2008). Conversely, hypermethylation of promoter regions of tumor suppressor genes (Greger et al., 1989; Sakai et al., 1991; Esteller, 2008) leads to loss of expression of key genes affecting pathways involved in maintenance of cellular functions, including cell cycle, apoptosis, and DNA repair (Esteller, 2007b). Several bona fide tumor suppressor genes are silenced by promoter hypermethylation in tumors. For instance, hypermethylation of the promoter of the DNA repair gene *hMLH1* is associated with early stages of endometrial and colon cancer, and microsatellite instability phenotype (Esteller et al., 1999). Epigenetically mediated silencing of cyclin-dependent kinase inhibitor 2A, which is crucial for control of cell cycle has been reported in several cancers (Brock et al., 2008; Liau et al., 2014). Additionally, pathways regulated by microRNAs have been associated with DNA hypermethylation-dependent silencing (Saito et al., 2006).

Besides methylating cytosines, DNMTs may coordinate other chromatin-mediated aspects of gene expression at sites of gene promoters (Herman and Baylin, 2003). For example, hypermethylation of promoters of tumor suppressor genes is associated with recruitment of proteins belonging to the methyl CpG-binding domain (MBD) family, MeCP2, MBD1, MBD2, MBD3, and MBD4 (Ballestar and Esteller, 2005). It has been shown that MeCP2 represses transcription of methylated DNA by recruiting histone deacetylases (HDACs), providing the first evidence for interactions between DNA methylation and histone modifications (Jones et al., 1998; Nan et al., 1998).

There is evidence that 1,25-D_3_ is able to induce DNA demethylation, however, the mechanisms behind the effect of 1,25-D_3_ on DNA methylation are not clear. In most cases it is probably passive demethylation that happens over several cycles of DNA replication. However, in some cases demethylation occurs within 1–4 h, indicative of an active process (Doig et al., 2013). The fact that vitamin D can alter methylation of DNA in the promoter of certain genes is novel. Tapp and colleagues suggested that in healthy subjects global, age-related CGI methylation of human rectal mucosa was influenced not only by gender, folate availability, and selenium, but also by vitamin D status (Tapp et al., 2013). The authors show negative association between serum 25-D_3_ level and CGI methylation of the adenomatous polyposis coli (*APC*) promoter region, a tumor suppressor often inactive in colorectal cancer. Interestingly, they observed a weak positive correlation of vitamin D level with methylation of *LINE-1* (genomic long interspersed nuclear element-1), a mammalian autonomous retrotransposon, increasing stability of this region (Tapp et al., 2013). A recent study in colorectal cancer patients investigating two Canadian populations (from Newfoundland and Ontario) found that high dietary vitamin D intake was associated with lower methylation of the two WNT antagonists *dickkopf 1* (*DKK1*) and *WNT5A* (Rawson et al., 2012). This relationship became even more significant in females in the Newfoundland population, while in the Ontario population the association between vitamin D intake and lower methylation was observed only in early stage tumors, but not in late stage tumors (Rawson et al., 2012). These data confer further insights in the mechanisms regulating the transcriptional activating effect of vitamin D on *DKK1* expression described *in vitro* (Aguilera et al., 2007; Pendas-Franco et al., 2008).

Moreover, treatment of the triple negative breast cancer cell line MDA-MB-231 with 1,25-D_3_ reduced DNA methylation of the *e-cadherin* promoter (Lopes et al., 2012), while another study showed that 1,25-D_3_ induced demethylation of the *PDZ-LIM* domain-containing protein 2 promoter, leading to increased expression (Vanoirbeek et al., 2014). In non-malignant and malignant prostate epithelial cells, treatment with 1,25-D_3_ caused clear changes in site-specific methylation of the *p21* promoter, in a cell line-specific manner (Doig et al., 2013).

An interesting interaction between vitamin D and DNA methylation is induction of the expression of GADD45 (growth arrest and DNA damage) protein by 1,25-D_3_ in several tumor cells (Jiang et al., 2003; Zhang et al., 2006; Bremmer et al., 2012). GADD45A is one of the enzymes that promote epigenetic gene activation by repair mediated DNA demethylation in Xenopus laevis (Barreto et al., 2007).

In summary, alterations in DNA methylation lead to aberrant gene expression and disruptions of genomic integrity, which contribute to development and progression of diseases. Vitamin D can regulate these processes; the mechanisms behind need further investigations.

### Interactions of vitamin D with chromatin modulators and remodelers

Nuclear receptors, such as the VDR contain DNA-binding domains that mediate binding to the DNA, presuming the DNA is available and is not wound tightly around nucleosomes. The chromatin context determines nuclear receptor binding and determines which epigenetic modifications will occur thereafter. Upon binding to their genomic response elements, nuclear hormone receptors will then recruit different regulatory cofactor complexes (Lee et al., 2001). The unliganded VDR is able to bind also genomic DNA, where it usually forms complexes with corepressor proteins that either exert HDAC activity, e.g., ALIEN (Polly et al., 2000), or are associated with HDACs, such as NCOR1 and SMRT. The corepressors dissociate upon binding of 1,25-D_3_, and are replaced by coactivator complexes.

The chromatin environment dictates gene activity throughout the genome. Post-translational modifications of the N-terminal tails of histone proteins allow nucleosomes to shift, the chromatin to relax, and genes to become activated. Histone modifications change in response to environmental stimuli (Meyer et al., 2013). Histones are major protein components of chromatin that undergo post-translational modifications, including acetylation of lysines, methylation of lysines and arginines, and phosphorylation of serine and threonine residues (Esteller, 2008). In epigenetically silenced genes, hypermethylation of CGIs is often associated with loss of acetylation on histone 3 and 4 (H3 and H4), loss of methylation of lysine (K) 4 on H3 (H3K4), and gain of methylation of K9 and K27 on H3 (H3K9 and H3K27) (Esteller, 2008).

Histone acetylation generally correlates with transcriptional activation (Hebbes et al., 1988; Kouzarides, 2007) and is dependent on a dynamic interaction between histone acetyltransferases (HATs) and histone deacetylases (HDACs) (Marks et al., 2001). The balance between the actions of these enzymes is crucial in controlling gene expression, and governs several developmental processes and disease states (Haberland et al., 2009). Generally, HATs are defined as activators of transcription, whereas HDACs as transcription repressors (Parbin et al., 2014). In various cancer types, including prostate, gastric, and breast cancers, overexpression of HDAC1 is often associated with poor clinical outcome (Choi et al., 2001; Halkidou et al., 2004; Zhang et al., 2005). In colorectal cancer patients HDAC1, 2, and 3 are overexpressed, and high HDAC1 and 2 expression is linked with reduced patient survival (Zhu et al., 2004; Wilson et al., 2006). Overexpression of HDAC1 plays a crucial role in regulating proliferation by repressing the expression of the cyclin-dependent kinase inhibitor p21 (Lagger et al., 2003). Additionally, silencing of HDAC4 leads to re-expression of p21, which in turn induces cell growth arrest and tumor growth inhibition, both *in vitro* and *in vivo* in a human glioblastoma model (Mottet et al., 2009). In addition to classical HDACs, another group of enzymes, the sirtuins (silent information regulator 2 proteins) are involved in histone deacetylation (Schwer and Verdin, 2008). Sirtuins have been linked to metabolic disorders, cancer, aging, and also regulation of the circadian rhythm (Guarente, 2006; Longo and Kennedy, 2006; Jung-Hynes et al., 2010).

**Figure 1:**
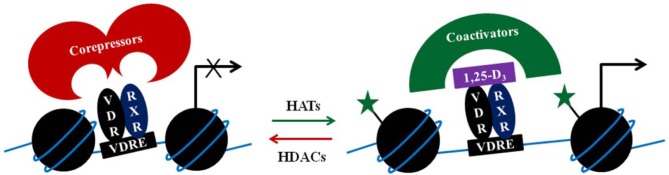
**Simplified illustration of a two-step coregulator model**. The VDR/RXR complex in absence of the 1,25-D_3_ may bind corepressors that would lead/maintain gene repression, partly by attracting histone deacetylases (HDACs). Upon ligand binding to the VDR/RXR complex, corepressors are replaced by coactivators, which include histone acetyltransferases (HATs). Acetylation of histones (indicated by the green stars) enables chromatin relaxation and gene transcription.

Many of the coactivators recruited by the VDR, including p160 steroid receptor coactivator proteins (SRC1, 2, and 3), p300, or CBP have lysine acetyltransferase activity (Figure 1). Indeed, treatment of THP-1 cells with 1,25-D_3_ increased H3K27ac at the promoter of several early VDR target genes (Seuter et al., 2013). In genetic hypercalciuric stone forming rats inhibition of bone morphogenetic protein 2 (BMP2) by 1,25-D_3_, seems to involve H3 deacetylation and H3K9 di-methylation (Fu et al., 2013).

In MDA-MB453 breast cancer cells 1,25-D_3_ treatment regulates expression of p21 through a mechanism involving both histone acetylation and methylation, probably by dynamic chromatin looping from distal 1,25-D_3_ responsive elements to the TSS of *p21* (Saramäki et al., 2009).

Histone methylation can lead either to gene activation or repression, depending on the histone site that is methylated, the degree of methylation (e.g., mono-methylation, di-methylation, or tri-methylation), amino acid residues affected, and their position in the histone tail (Esteller, 2008). Methylation of histones depends on a dynamic process arising from the actions of methyltransferases (HMTs) and demethylases (HDMs) (Shi and Whetstine, 2007; Mosammaparast and Shi, 2010; Greer and Shi, 2012). So far, two protein families capable of demethylating lysines are known, the amine oxidases (Shi et al., 2004) and jumonji C (JmjC)-domain-containing proteins (Cloos et al., 2006; Tsukada et al., 2006). The first histone demethylase discovered was the lysine-specific demethylase 1 (LSD1/KDM1A), an amine oxidase, which demethylates H3K4me2/me1 (Table 1) (Shi et al., 2004). High expression of KDM1A in various cancers, including colorectal cancer, prostate cancer, and neuroblastomas is associated with increased cancer recurrence and poor clinical outcome (Kahl et al., 2006; Schulte et al., 2009; Ding et al., 2013). Additionally, it has been shown that LSD1 is essential for androgen and estrogen receptor-dependent gene activation via H3K9me2/me1 demethylation (Metzger et al., 2005; Garcia-Bassets et al., 2007; Perillo et al., 2008). There is a reciprocal regulatory effect between the activity of VDR and histone demethylases. In the colon cancer cell line SW480-ADH 1,25-D_3_ increased the expression of the lysine-specific demethylase 1 and 2 (Pereira et al., 2012).

**Table 1:** **A simplified list of the members of the two classes of histone demethylases (mentioned in the manuscript)**.

**Class of histone demethylases**	**Histone demethylase family**	**Histone demethylase**	**Histone substrate**	**Gene expression**
Amine oxidases	KDM1	KDM1A	H3K4me2/me1	Repression
			H3K9me2/me1	Activation
		KDM1B	H3K4me2/me1	Repression
Jumonji C-domain-containing proteins	KDM2	KDM2A	H3K36me2/me1	Repression
		KDM2B	H3K4me3	
			H3K36me2/me1	
	KDM3	KDM3A	H3K9me2/me1	Activation
		KDM3B	H3K9me3/me2/me1	
	KDM4	KDM4A	H3K9me3/me2	Activation
		KDM4B	H3K36me3/me2	Repression
		KDM4C		
		KDM4D	H3K9me3/me2	Activation
	KDM5	KDM5A	H3K4me3/me2	Repression
		KDM5B		
		KDM5C		
		KDM5D		
	KDM6	KDM6A	H3K27me3/me2	Activation
		KDM6B		
	PHF	JHDM1D	H3K9me2/me1	Activation
			H3K27me2/me1	
		PHF8	H3K9me2/me1	

Reviewed in Pedersen and Helin (2010), Greer and Shi (2012). The main histone demethylase families and submembers are indicated. Degree of methylation and site of lysine residue are given. References for individual enzymes can be found throughout the text. H, Histone; K, lysine; me1, mono-methylation; me2, di-methylation; me3, tri-methylation; KDM1A, lysine-specific demethylase 1A; JHDM1D, JmjC-domain-containing histone demethylation protein 1D; PHF, plant homeodomain finger protein.

1,25-D_3_ treatment affected also the expression of a series of different JmjC histone demethylases. The first identified member of the JmjC family was KDM2A/JHDM1A (Tsukada et al., 2006). Expression profiling data showed altered expression of KDM2A and KDM2B in several tumors, however, it seems that their pro- or antioncogenic functions are tissue-dependent (Frescas et al., 2007, 2008; Pfau et al., 2008). 1,25-D_3_ inhibited the expression of several histone demethylases (e.g., KDM4A/4C/4D/5A/2B, JMJD5/6, PLA2G4B), and induced the expression of others, JARID2 and KDM5B (Pereira et al., 2012). Members of the KDM4 family catalyze tri-demethylation of H3K9 and/or H3K36 (Cloos et al., 2006; Fodor et al., 2006; Klose et al., 2006; Whetstine et al., 2006; Lin et al., 2008). H3K9me3 is a mark for heterochromatin and demethylation of H3K9 is suggested to be linked with chromosomal instability (Cloos et al., 2006). Inhibition of expression of KDM4 family members by 1,25-D_3_ could thus contribute to genome stability. Members of KDM5 cluster catalyze demethylation of H3K4me3/me2, which is a mark for open chromatin (Christensen et al., 2007; Iwase et al., 2007; Klose et al., 2007; Tahiliani et al., 2007) and their upregulaton upon 1,25-D_3_ treatment might lead to gene repression (Pereira et al., 2011). Overexpression of KDM5B has been reported in breast and prostate cancers (Lu et al., 1999; Xiang et al., 2007). Deletion of *kdm5b* inhibits tumor growth in a syngeneic mouse mammary tumor (Yamane et al., 2007), suggestive of its potential role in tumor development. 1,25-D_3_ induced the expression of the histone demethylase KDM6B as well, which is the only other known enzyme, besides KDM6A that is able to demethylate H3K27me3, a histone mark that correlates with gene repression. Furthermore, the authors showed positive correlation between KDM6B and VDR in 96 colon tumor patients, and inverse correlation of KDM6B with SNAIL1, which is involved in epithelial to mesenchymal transition, indicating that probably the antiproliferative role of 1,25-D_3_ via KDM6B upregulation might take place *in vivo* (Pereira et al., 2011). Interestingly, treatment of SW480-ADH cells with 1,25-D_3_ had no effect on global H3K27me3 levels, in spite of KDM6B upregulation (Pereira et al., 2011, 2012). The effect of 1,25-D_3_ on the expression of histone demethylases may well be indirect and could be mediated by microRNAs (Padi et al., 2013). KDM2A is one of the direct targets of microRNA-627. 1,25-D_3_-dependent upregulation of the microRNA-627 expression both *in vitro*, in the HT-29 colorectal cancer cells and *in vivo*, in tumor xenografts, led to lower KDM2A levels (Padi et al., 2013).

In different pathologies, the expression pattern of the nuclear receptor cofactors is altered, compromising the effect of 1,25-D_3_ (Doig et al., 2013; Singh et al., 2013). The initial interactions between VDR and coactivators are the seed for the assembly of intricate multiprotein complexes that remodel the chromatin structure, recruit the core transcriptional machinery, and induce expression of 1,25-D_3_ target genes (Figure 1). Often, differences in responsiveness to 1,25-D_3_ depend on the expression pattern of the coregulators of VDR. In prostate cancer cells, the temporal distribution of the nuclear corepressor NCOR1 at VDR target genes is different in 1,25-D_3_ responsive cells compared with unresponsive cells (Doig et al., 2013; Singh et al., 2013).

The liganded VDR is able both to transactivate and transrepress target genes. The mechanisms of action are probably different between transactivation and transrepression, and also highly dependent on the motifs of the vitamin D response elements. A highly complex mechanism regulates the ligand-dependent repression of CYP27B1 (Kim et al., 2007a). CYP27B1 repression requires two epigenetic modifications: deacetylation of histones and methylation of the *CYP27B1* gene promoter and exon regions. This is dependent on the presence of the VDR interacting repressor (VDIR) and the chromatin remodeler Williams Syndrome transcription factor. In the absence of 1,25-D_3_, VDIR is bound directly to the E-box motifs in the negative VDRE and recruits histone acetyltransferases to induce *CYP27B1* gene transcription. In the presence of 1,25-D_3_, VDIR acts as a scaffold for the 1,25-D_3_-VDR complex to repress transcription of *CYP27B1* through recruitment of HDAC2, DNMT1, and DNMT3B (Kim et al., 2007a). It seems that VDIR and HDAC2 are involved also in the 1,25-D_3_-dependent transrepression of the human parathyroid hormone gene (Kim et al., 2007b). It is not clear, whether this mechanism of transrepression by liganded VDR also applies to other genes. In mesenchymal stem cells 1,25-D_3_ represses gene expression by binding to promoters with enhanced H3K9Ac and H3K9me2 (Tan et al., 2009). Whether H3K9 acetylation/methylation enabled VDR binding or VDR binding caused H3K9 acetylation, is not clear.

Proper orchestration of histone modifications in crosstalk with other chromatin regulators is crucial in maintaining the epigenetic landscape and governing gene expression. Any disturbances in these constellations may lead to aberrant gene expression. Whether 1,25-D_3_ affects regulation of other chromatin modulators as well, is not yet known.

## Regulation of the vitamin D system

The vitamin D system has pleiotropic functions and regulates approximately 3% of the human genome (Bouillon et al., 2008). To maintain balance, a strict regulation of the vitamin D system genes is of utmost importance. The main role of liganded VDR in tissues not involved in calcium homeostasis is to control expression of genes that regulate cell proliferation, differentiation, and apoptosis. One major limitation in the therapeutic exploitation of these effects is the resistance of cancer cells to 1,25-D_3_. Epigenetic corruption of VDR signaling is suggested to be one of the mechanisms that leads to reduced responsiveness to 1,25-D_3_ actions. This can be caused by promoter methylation of key vitamin D system genes or by skewed accumulation of VDR-associated co-repressors, preferentially at promoters of anti-proliferative target genes (Abedin et al., 2006).

**Figure 2:**
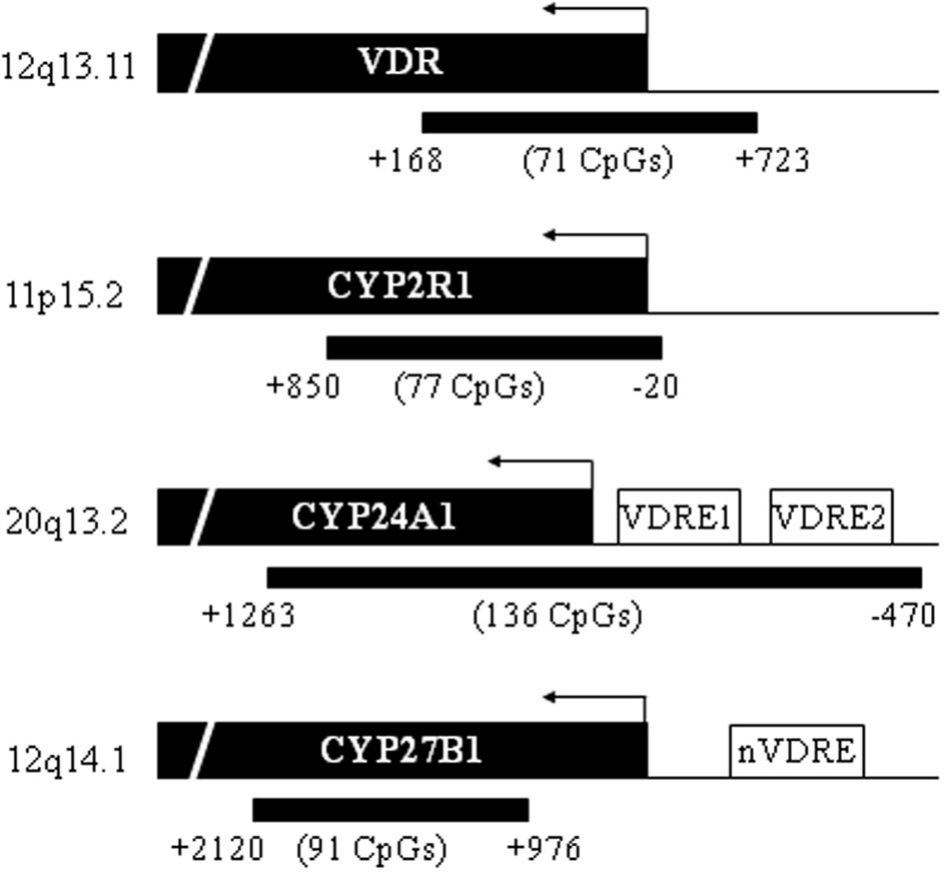
**Location of CpG islands in the promoter region of vitamin D tools genes**. VDR is located on 12q13.11 (chr12:48235320-48298814), CYP2R1 on chromosome 11p15.2 (chr11:14899556-14913751), CYP24A1 on chromosome 20q13.2 (chr20:52769988-52790516), and CYP27B1 on 12q14.1 (chr12:58156117-58160976). The locations of the CpG islands are indicated (black bars) relative to start of the gene locus (not to TSS) according to UCSC Genome Browser Homepage (GRCh37/hg19) (Karolchik et al., 2014). Number of CpGs located within each island is stated. Two vitamin D responsive elements (VDRE) are located in the proximal CYP24A1 promoter region and one nVDRE is located in the CYP27B1 promoter region.

Expression of the vitamin D degrading and metabolizing enzymes is regulated through binding of 1,25-D_3_-liganded VDR to vitamin D responsive elements (VDREs). However, the major regulators of 1,25-D_3_ levels and signaling CYP2R1, CYP24A1, CYP27B1, and VDR, “the vitamin D tool” genes, are prone to epigenetic regulation. CpG islands span the promoters of *CYP2R1*, *CYP24A1*, and *VDR*, while a CpG island is located within the *CYP27B1* gene locus (Figure 2). Therefore, DNA methylation and histone modifications in these regions can change the chromatin state from an open to closed conformation and lead to transcriptional repression of these genes. Expression of vitamin D tool genes becomes deregulated in various types of cancer, and these changes may be partially attributed to epigenetic alterations (reviewed in Hobaus et al., 2013). As early as 1984, Yoneda et al. (1984) have shown that the histone acetyltransferase inhibitor butyrate augments 1,25-D_3_ actions. Several studies confirmed these findings (e.g., Rashid et al., 2001) and have suggested that the action of butyrate could be through upregulation of VDR or CYP27B1 expression (Gaschott and Stein, 2003). Whether this effect is mediated by direct acetylation of the *VDR* or *CYP27B1* promoters, has not been determined.

In this chapter we discuss evidence for epigenetic regulation through DNA methylation of these genes in health and disease.

### Epigenetic regulation of the vitamin D receptor

The VDR is a nuclear receptor mediating 1,25-D_3_ signaling. It is expressed by at least 38 cell types in the human body (Norman and Bouillon, 2010). In the absence of its ligand 1,25-D_3_, VDR is mainly found in the cytoplasm (Nagpal et al., 2005). Upon ligand binding, VDR heterodimerizes with the retinoid X receptor (RXR) and translocates to the nucleus, where it binds to vitamin D responsive elements (VDREs) to regulate transcription of 1,25-D_3_ target genes. This is achieved through recruitment of coactivators or corepressors to the VDR-RXR complex bound to DNA (Nagpal et al., 2005; Pike et al., 2012; Haussler et al., 2013; Pike and Meyer, 2013). As VDR is rarely mutated during carcinogenesis (Miller et al., 1997), the disturbance of the vitamin D signaling and apparent 1,25-D_3_ insensitivity in cancer (Marik et al., 2010) must be attributed to other alterations, which may include epigenetic changes, such as promoter methylation.

The *VDR* gene is located on the long arm of chromosome 12 (12q13.11) and contains 4 potential promoter regions. Exons 1a, 1c, and 1d of the *VDR* are well conserved, while 1b, 1e, and 1f show low homology (Halsall et al., 2007). Exon 1a appears to contain a strong promoter, including several transcription factor binding sites (AP-2 and SP1). Transcription was reported to originate also in exons 1d, 1e, and 1f, while translation starts in exon 2 (Halsall et al., 2007). Marik and colleagues performed an *in silico* analysis of the *VDR* gene sequence and reported three CpG islands located in exon 1a spanning from −790 bp to 380 bp relative to the TSS in exon 1a (Marik et al., 2010). According to the UCSC Genome browser, however, only one large CpG island spanning 892 bp in length is found in the *VDR* promoter region (Gardiner-Garden and Frommer, 1987; Karolchik et al., 2014). This discrepancy is likely due to the different search parameters used for CpG island identification.

Epigenetic silencing of VDR was suggested to cause the slow normalization of VDR levels in the parathyroid glands of uremic rats after kidney transplantation (Lewin et al., 2002; Hofman-Bang et al., 2012). However, sequencing of the *VDR* promoter [−250 to 300 bp relative to exon 1 (43 CpGs)] in normal and uremic rats showed no difference between methylation patterns (Hofman-Bang et al., 2012). Further, the authors reported that methylation levels coincided with the negative control, thus showing that promoter methylation does not play a role in regulating VDR expression in the parathyroid glands.

In contrast, promoter methylation was reported to cause repression of *VDR* gene expression in HIV infected T cells. In normal T cells, activation or priming causes an upregulation of VDR expression (Von Essen et al., 2010). In comparison, infection of previously activated T cells with human immunodeficiency virus (HIV) led to upregulation of DNMT3B, increased promoter methylation of *VDR* (45–70%), and decreased *VDR* gene expression (Chandel et al., 2013). This downregulation of VDR could be reversed upon treatment with 5-azacytidine (AZA) suggesting that the decreased expression of VDR by HIV is, at least partially, caused by DNA methylation (Chandel et al., 2013). There is evidence for an inverse correlation between the vitamin D status and infections, however, many trials failed to show a protective effect of vitamin D (reviewed in Yamshchikov et al., 2009). Thus, reduced sensitivity to vitamin D metabolites due to, e.g., downregulation of VDR may account for inconclusive trials. This is supported by a study investigating methylation of the 3′end of *VDR* in two South African groups revealing differences with respect to ethnicity and tuberculosis status of the patients (Andraos et al., 2011).

In breast tumors, methylation of exon 1a of the *VDR* gene was significantly higher (65% of CpGs methylated) compared with normal breast tissue (15% of CpGs methylated) (Marik et al., 2010). *In vitro*, in breast cancer cell lines, three hypermethylated regions in exon 1a became demethylated after treatment with the DNMT1 inhibitor 5-aza-2′-deoxycytidine (DAC) and VDR mRNA expression increased. These regions were in proximity to the SP1 binding sites (approximately 790 bp from TSS), NFκ B binding sites (approximately −480 from TSS), and the exon 1a TSS. Treatment with 1,25-D_3_ had no effect on methylation of these regions (Marik et al., 2010). In other types of cancer, e.g., the choriocarcinoma-derived trophoblast cell lines JEG-3 and JAR, the *VDR* promoter was densely methylated (Novakovic et al., 2009). In contrast, no methylation of the VDR promoter region was observed in colon cancer cell lines, and treatment with DAC did not increase gene expression (Habano et al., 2011; Höbaus et al., 2013a). In parathyroid tumors the expression of VDR is decreased (Gogusev et al., 1997; Carling et al., 2000), however, no differences in DNA methylation of *VDR* were observed between parathyroid tumors and healthy controls (Sulaiman et al., 2013). Similar results were seen in parathyroid adenoma samples, which showed decreased expression of VDR, but showed no promoter methylation (Varshney et al., 2013).

Additionally, it has been suggested that expression of 5′ truncated variants of VDR is linked to methylation of the *VDR* promoter. These variants are predominantly found in breast cancer compared with the full length variants expressed in normal breast tissue. Treatment with DAC restored expression of the active transcript variant of VDR in breast cancer cell lines, indicating promoter methylation as cause of truncated protein expression (Marik et al., 2010). The significance of these potentially untranslated truncated variants remains to be investigated, however, as they are not found in normal breast tissue aberrant expression of truncated isoforms may further disrupt vitamin D signaling in tumor tissue.

A recent study suggested that in colorectal cancer metastases, VDR becomes the target of the polycomb group protein enhancer of zeste homolog 2 (EZH2) that mediates VDR downregulation by H3K27 trimethylation in the VDR promoter (Lin et al., 2013). The histone deacetylase HDAC3, one of the most frequently upregulated genes in cancer, seems to inhibit VDR expression. In two colorectal cancer cell lines, HCT116 and SW480 knock down of HDAC3 increased VDR expression and restored sensitivity of these cells to 1,25-D_3_ (Godman et al., 2008).

Taken together, there is evidence that in various diseases the decreased tissue sensitivity to 1,25-D_3_ could have been caused by the epigenetic silencing of the VDR.

### Epigenetic regulation of the CYP2R1

CYP2R1 is a microsomal P450 enzyme, which hydroxylates both vitamin D_2_ and D_3_ at position C-25 to form the circulating storage form of vitamin D 25-D_3_. The promoter region of *CYP2R1* is located within a CpG island, which can be subjected to epigenetic regulation. So far, only two studies investigated the promoter methylation status of this gene. Genome wide association studies found increased *CYP2R1* promoter methylation in leukocyte DNA from individuals with severe vitamin D deficiency compared with control group (Zhu et al., 2013). Further, methylation levels of *CYP2R1* promoter decreased within 12 months of vitamin D supplementation in DNA extracted from serum of non-Hispanic white American post-menopausal women aged ≥55 years (Zhu et al., 2013), indicating an effect of vitamin D supplementation on *CYP2R1* promoter methylation. These data indicate that under low vitamin D serum levels, the promoter of the major 25-hydroxylase *CYP2R1* may become methylated, and that event appears to be reversible upon exposure to increased vitamin D.

### Epigenetic regulation of the CYP27B1

CYP27B1 is an inner mitochondrial membrane P450 enzyme that converts 25-D3 to its active form 1,25-D3. It is mainly expressed in the proximal tubule of the kidneys, but it is also expressed in many vitamin D target tissues, albeit at lower levels (Hendrix et al., 2004). The *CYP27B1* gene harbors a CpG island. However, recent sequence updates (Ensembl 74, November 2013) shift the CpG island from the *CYP27B1* promoter region into the gene coding sequence (Flicek et al., 2014). This explains the differences between the location of the CpG island depicted in Figure 2 and the location of the CpG island described in literature. For simplicity, statements on nVDRES and CpG island location below refer to reports in the published articles and not to Figure 2.

The promoter region of *CYP27B1* contains a negative VDRE (nVDRE) located at around 500 bp, consisting of two E-box like motifs (Murayama et al., 2004). This region is responsible for 1,25-D_3_-dependent transrepression, which seems to be achieved through recruitment of both HDACs and DNMTs by VDR/RXR to the promoter region of *CYP27B1* (Takeyama and Kato, 2011). For further details see subsection Interactions of Vitamin D with Chromatin Modulators and Remodelers.

In cancer, expression of CYP27B1 is often downregulated. This may be explained by increased methylation of the CpG island located within *CYP27B1*. In the breast cancer cells MDA-MB-231, *CYP27B1* hypermethylation led to gene silencing, which could be reversed by treatment with deoxyC (Shi et al., 2002). In prostate cancer cell lines, combination of the DNMT1 inhibitor DAC and the HDAC inhibitor TSA resulted in increased activity of CYP27B1 (Khorchide et al., 2005). In the choriocarcinoma cell lines BeWo and JAR the promoter of *CYP27B1* was densely methylated (Novakovic et al., 2009). The *CYP27B1* promoter was hypermethylated (61%) in Non-Hodgkin's lymphoma, but not in benign follicular hyperplasia. Two out of four non-Hodgkin's lymphoma cell lines showed strong methylation of the *CYP27B1* promoter. Interestingly, all four responded to DAC-TSA treatments with upregulation of gene expression independent of the methylation status of their *CYP27B1* promoter, which may be explained by other regions prone to methylation not investigated in this study or by differences in silencing mechanisms (Shi et al., 2007). Further, the methylation level of *CYP27B1* was increased in primary lymphoma and leukemia cells also compared with normal peripheral blood lymphocytes (Lagger et al., 2003; Wjst et al., 2010).

Methylation of *CYP27B1* in diseases might cause reduced local activation of 25-D_3_ to 1,25-D_3_, thus reducing local 1,25-D_3_ levels and restricting its functions.

### Epigenetic regulation of CYP24A1

The 1,25-dihydroxyvitamin D_3_ 24-hydroxylase is an inner mitochondrial membrane P450 enzyme, which catalyzes both 25-D_3_ and 1,25-D_3_ (Kawashima et al., 1981; Pedersen et al., 1983; Sakaki et al., 2000). Its primary site of expression are the kidneys, where it plays a crucial role in regulating systemic vitamin D metabolite levels, however, expression is found in many other vitamin D target tissues.

The promoter of *CYP24A1* is spanned by a CpG island making it prone to regulation by DNA methylation. Several responsive elements are located within this area, including two VDREs, a vitamin stimulating element (VSE), and SP1 binding sites (see Figure 2).

In healthy kidney, skeletal muscle, whole blood, brain, skin fibroblasts, and sperm the *CYP24A1* promoter is not methylated (Novakovic et al., 2009), although the expression levels are highly variable. In peripheral blood lymphocytes methylation of *CYP24A1* was low (5%) (Wjst et al., 2010). Interestingly, in full term human placenta 56.5% of the *CYP24A1* promoter is methylated. *CYP24A1* methylation was also observed in the placenta of the marmoset and mouse, however, at a lower level.

In the choriocarcinoma cell lines JEG-3, BeWo, and JAR the promoter of *CYP24A1* was densely methylated and the methylation level correlated inversely with the low gene expression (Novakovic et al., 2009). Treatment of osteoblastic ROS cells with 1,25-D_3_ did not induce CYP24A1 expression. Considering the strong methylation of the *CYP24A1* promoter region, epigenetic silencing of CYP24A1 may account for the unresponsiveness of this gene to 1,25-D_3_ (Ohyama et al., 2002). In the human prostate cancer cell line PC3, methylation of the *CYP24A1* promoter reduced reporter gene expression in a methylation-dependent manner (Luo et al., 2010). In prostate cancer cells, the methylation status of *CYP24A1* promoter inversely correlated with gene expression. Demethylating agents restored CYP24A1 expression only in cell lines where the *CYP24A1* promoter was methylated prior to treatment (Luo et al., 2010). Only DNA demethylation by DAC treatment permitted recruitment of VDR to the *CYP24A1* promoter (Luo et al., 2010). In patients, development from benign toward malignant prostate lesions was paralleled by increasing methylation levels of the *CYP24A1* promoter (Luo et al., 2010). Prostate tumor derived endothelial cells (TDEC) expressed less CYP24A1 compared with endothelial cells derived from normal cells or matrigel plugs, which may be attributed to increased *CYP24A1* promoter methylation in TDECs (Johnson et al., 2010). We have shown recently that in colon cancer cell lines DAC induced CYP24A1 expression in a cell line-specific manner, independent of the methylation level of the promoter. In these cells induction of CYP24A1 expression by DAC seems to be independent of *CYP24A1* promoter methylation (Höbaus et al., 2013a). Moreover, the methylation level of the *CYP24A1* promoter was comparably low both in colon adenocarcinomas and the adjacent mucosa, although the expression of CYP24A1 was significantly higher in the tumors (Höbaus et al., 2013b).

Taken together, the regulation of CYP24A1 by DNA methylation appears to be tissue-dependent, both in health and disease.

## Conclusions

There is a strong reciprocity between the vitamin D system and epigenetic mechanisms. The vitamin D system is, on the one hand regulated by epigenetic mechanisms and, on the other hand, is involved in regulating epigenetic events. Critical vitamin D tool genes can be silenced by DNA methylation. The VDR protein interacts, directly or indirectly, with chromatin modifiers and remodelers. Liganded VDR regulates expression of several of these chromatin modifiers and remodelers, and it might even regulate DNA methylation.

Epigenetic regulation of gene expression is a fine-tuned mechanism and its deregulation can lead to pathological conditions. The impact of vitamin D in the maintenance of the normal epigenetic landscape underlines the central role of this hormone in physiology.

## Perspectives

One of the most fundamental questions in the control of gene expression is the way how epigenetic marks are established, erased, and recognized. Regulating epigenetic events could be a further mechanism by which 1,25-D_3_ may prevent or delay tumorigenesis and onset of chronic diseases. Therefore, we need to understand better the impact of vitamin D on the epigenome, and plan thorough and comprehensive studies to examine this interplay.

### Conflict of interest statement

The authors declare that the research was conducted in the absence of any commercial or financial relationships that could be construed as a potential conflict of interest.
